# Early mineralocorticoid receptor antagonism for end-organ protection in hypertension: Implications for disparities and rethinking the timing paradigm

**DOI:** 10.1016/j.dialog.2026.100343

**Published:** 2026-08-19

**Authors:** Camellus O. Ezeugwu

**Affiliations:** Johns Hopkins University, Baltimore, MD, USA; Just Heart Cardiovascular Group Inc., MD, USA; Heart Disease Prevention & Training Center (HDPTC) Inc., USA

**Keywords:** Hypertension, Aldosterone, Mineralocorticoid receptor antagonists, End-organ damage, Health disparities, Finerenone, Hyperkalemia

## Abstract

**Purpose:**

Hypertension-related end-organ damage disproportionately affects Black populations, with earlier onset, greater severity, and higher rates of heart failure and chronic kidney disease despite treatment. Because structural injury may persist even when blood-pressure (BP) targets are met, this review examines aldosterone–mineralocorticoid receptor (MR) signaling as a driver of residual tissue injury and develops a hypothesis-generating framework for earlier, risk-stratified MR antagonism.

**Methods:**

This is a narrative review. We searched PubMed/MEDLINE and the Cochrane Library (inception–July 2026) using terms including mineralocorticoid receptor antagonist, aldosterone, spironolactone, eplerenone, finerenone, hypertension, resistant hypertension, end-organ damage, fibrosis, hyperkalemia, chronic kidney disease, heart failure, and health disparities. We prioritized randomized controlled trials, prespecified pooled analyses, mechanistic studies, and current guidelines, and hand-searched reference lists. No quantitative synthesis was performed.

**Findings:**

Aldosterone–MR signaling promotes fibrosis, inflammation, oxidative stress, and adverse remodeling across cardiovascular and renal tissues, and may be particularly relevant in low-renin, salt-sensitive phenotypes observed more frequently, on average, among Black patients. Randomized trials support MR antagonists (MRAs) in resistant hypertension, heart failure, and diabetic CKD; however, MRAs are often introduced late, when reduced renal function raises hyperkalemia risk and limits preventive benefit.

**Conclusion:**

In selected higher-risk patients with preserved renal function and controlled potassium, earlier MR antagonism may warrant prospective evaluation as a tissue-protective, disparity-reducing strategy. This framework is hypothesis-generating and is intended to stimulate research, not to alter current guideline-directed therapy.

## Graphical abstract

Hypertension-related end-organ injury may progress despite blood pressure control due to persistent aldosterone–mineralocorticoid receptor signaling. Earlier, risk-stratified mineralocorticoid receptor antagonism—before advanced renal dysfunction—may better prevent tissue injury while minimizing hyperkalemia risk.

**Figure:**
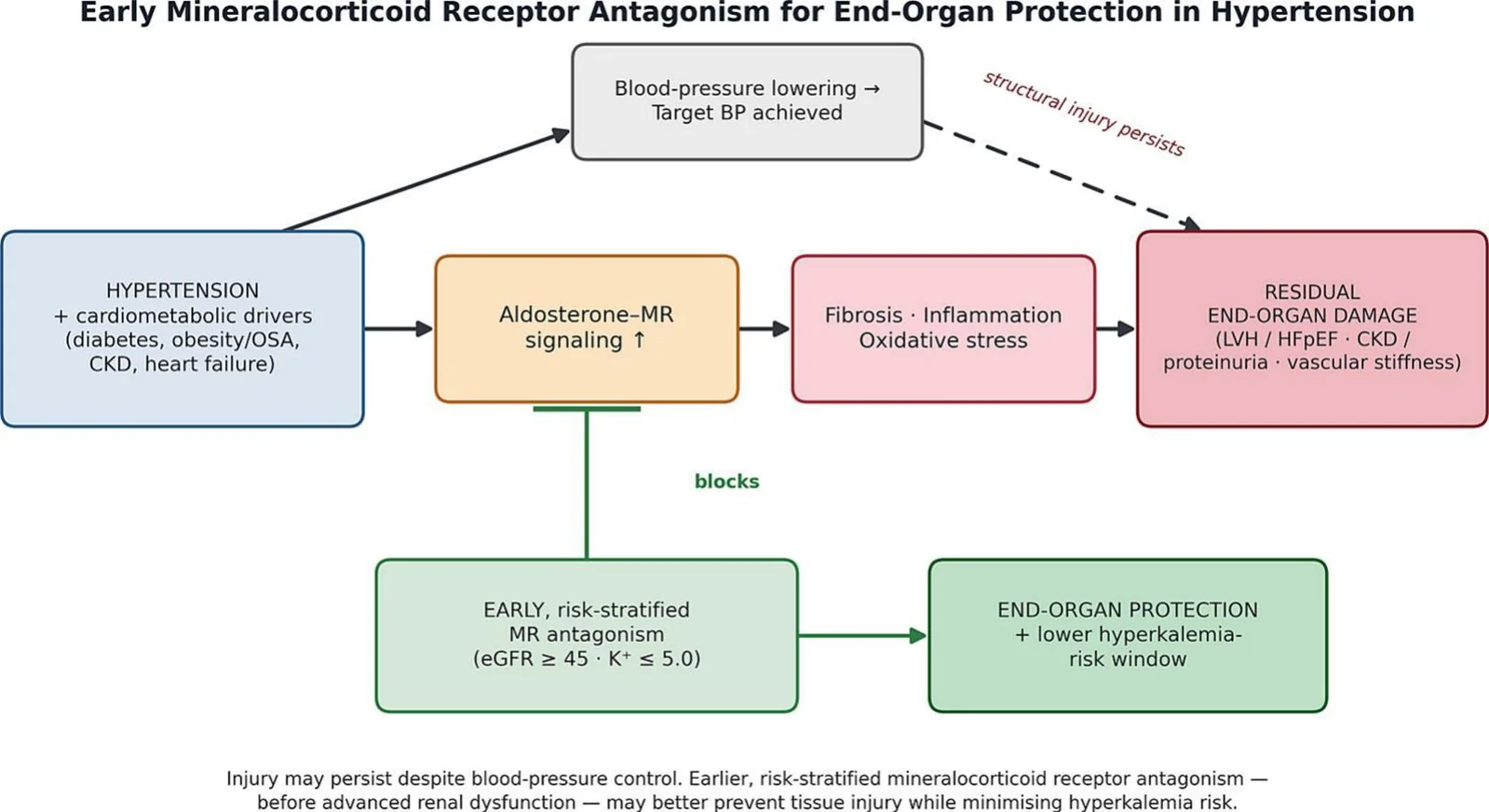

## 1 Introduction: BP control is necessary but not sufficient

Hypertension remains a leading driver of cardiovascular and renal morbidity. Guidelines appropriately emphasize BP reduction to reduce stroke, myocardial infarction, and heart failure risk [1], [2].

**Fig. 1:**
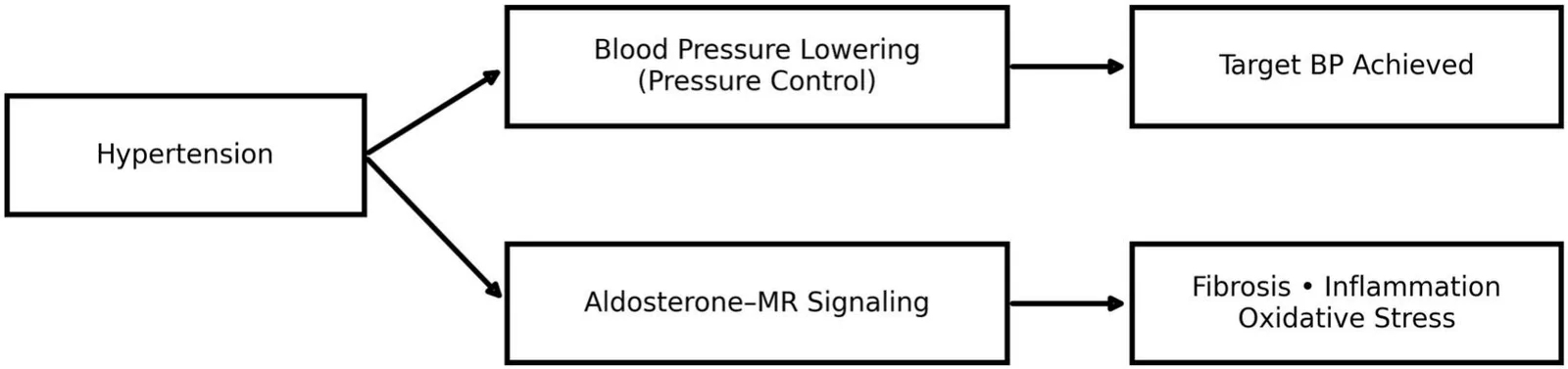
Pressure control versus tissue protection. Blood pressure lowering reduces mechanical stress but does not fully attenuate aldosterone–mineralocorticoid receptor–mediated tissue injury that drives fibrosis and inflammation.

Landmark trials such as SPRINT and STEP demonstrated that intensive BP lowering reduces major cardiovascular events [3], [4]. However, residual cardiovascular and renal risk persists even among intensively treated patients, and structural remodeling—including LV hypertrophy, HFpEF development, atrial remodeling, and albuminuria progression—may occur despite apparent BP control. These observations suggest that hypertension is increasingly recognized as both a hemodynamic disorder and a biologically mediated condition characterized by maladaptive neurohormonal activation and tissue remodeling. A pragmatic end-organ protection framework therefore pairs BP lowering with interventions that directly attenuate maladaptive biologic signaling (Fig. 1).

## 2 Evidence identification and synthesis (narrative review methods)

This is a narrative review synthesizing mechanistic and clinical evidence on aldosterone–MR signaling and MR antagonism in hypertensive end-organ protection. We searched PubMed/MEDLINE and the Cochrane Library from inception through July 2026, combining the terms mineralocorticoid receptor antagonist, aldosterone, spironolactone, eplerenone, finerenone, hypertension, resistant hypertension, end-organ damage, fibrosis, hyperkalemia, chronic kidney disease, heart failure, health disparities, and Black/African American. We prioritized randomized controlled trials, prespecified pooled analyses, mechanistic and translational studies, and contemporary guideline statements, and hand-searched the reference lists of key articles. English-language human studies were emphasized, with seminal preclinical work included where mechanistically relevant. Consistent with the conceptual, hypothesis-generating aim of this review, no formal systematic-review protocol (e.g., PRISMA) or quantitative synthesis was undertaken; source selection reflected relevance to the questions addressed and is subject to the limitations inherent to narrative reviews, including potential selection bias.

### 2.1 Residual organ injury despite blood pressure control

Importantly, observational and imaging data suggest that structural organ injury may persist even among patients achieving contemporary BP targets. Regression of left ventricular hypertrophy may plateau, albuminuria may persist, and vascular stiffness may progress despite adequate numeric BP control [3], [4], [5], [6]. These findings support the concept that hypertension-related injury reflects both hemodynamic load and sustained neurohormonal signaling [7], [8], [9]. A biologically informed therapeutic strategy may therefore complement pressure reduction with targeted modulation of tissue-level remodeling pathways.

### 2.2 Hypertension disparities and aldosterone biology

Hypertension prevalence and complications are disproportionately higher among Black adults in the United States, with earlier onset and increased risk of stroke, heart failure, and chronic kidney disease. These disparities are driven predominantly by social determinants of health, structural inequities, and differential access to care, and should not be attributed to race as a biological category. Within this context, one contributing and heterogeneous biological factor is the observation that low-renin and salt-sensitive phenotypes are, on average, more frequently identified in populations of African ancestry [10], [11]. Importantly, there is substantial heterogeneity within this population, and these phenotypes are neither universal nor deterministic; individual renin/aldosterone profiling—rather than race-based assumptions—should guide any biological interpretation [10]. Where present, a low-renin, salt-sensitive profile may be associated with heightened aldosterone activity and MR signaling, providing a mechanistic rationale for considering MR-directed therapy as one component of a comprehensive end-organ-protection strategy [12], [13]. MR antagonists nonetheless remain underused in earlier disease, where preventive potential may be greatest.

### 2.3 The expanding spectrum of primary aldosteronism

These observations align with an evolving understanding of primary aldosteronism (PA) as a broad, continuous spectrum rather than a rare, categorical disorder. Contemporary data indicate that autonomous or dysregulated aldosterone production is considerably more common than historically recognized, extending below classic diagnostic thresholds and across the blood-pressure range—from normotension to resistant hypertension [14], [15]. A suppressed or low plasma renin, particularly when accompanied by an aldosterone concentration that is inappropriate relative to renin and sodium status, is increasingly recognized as a marker of mineralocorticoid excess physiology and may identify patients in whom MR-directed therapy is particularly rational [14]. Because low-renin, salt-sensitive physiology is, on average, more frequent among Black adults, this expanded PA-spectrum framework further supports the consideration of earlier MR antagonism as part of a biologically informed, disparity-reducing strategy, while recognizing that formal PA case detection should continue to follow established diagnostic pathways [16].

### 2.4 Why aldosterone matters: beyond sodium retention, a common effector for *adverse tissue injury*

**Fig. 2:**
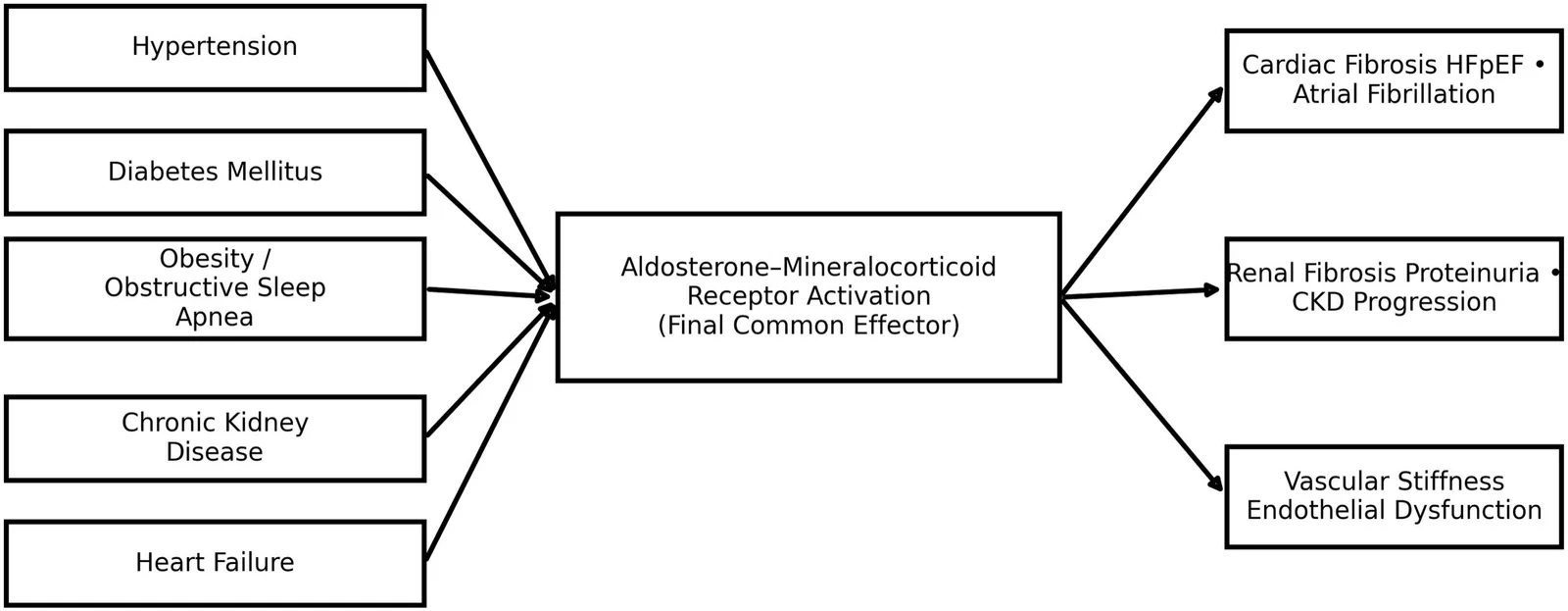
Clinical convergence pathways into aldosterone-mediated injury. Diverse cardiometabolic conditions converge on aldosterone–mineralocorticoid receptor activation, resulting in shared downstream cardiac, renal, and vascular phenotypes.

Aldosterone's canonical renal effects—sodium retention and potassium excretion—contribute to volume-dependent hypertension and treatment resistance [17], [18]. However, mineralocorticoid receptors are widely expressed in vascularized tissues, enabling direct cardiac, renal, vascular, and immune effects [7], [8]. Chronic diseases such as hypertension, obesity/OSA, diabetes, CKD, and heart failure each increase RAAS tone and/or aldosterone activity [13], [19], [20]. Despite differing upstream triggers, downstream phenotypes converge, supporting aldosterone–MR signaling as a key convergent mediator and systems-level amplifier of tissue injury that promotes fibrosis, inflammation, and oxidative stress across cardiac, renal, and vascular tissues (Fig. 2).

Chronic MR activation promotes:

• Fibroblast activation
• Extracellular matrix deposition
• Oxidative stress
• Pro-inflammatory signaling
• Endothelial dysfunction

Importantly, aldosterone escape or breakthrough can occur during ACE inhibitor or ARB therapy [21], [22]. Thus, downstream MR-mediated tissue injury may persist despite upstream RAAS blockade.

### 2.5 Ubiquity of MR expression and systems-level tissue injury

In humans, mineralocorticoid receptors extend well beyond renal epithelial cells and are broadly expressed across cardiovascular, renal, metabolic, and immune cell types, permitting aldosterone signaling to drive direct profibrotic, proinflammatory, and pro-oxidative tissue effects independent of classical sodium-retention pathways. MRs are present in cardiomyocytes, cardiac fibroblasts, endothelial cells, vascular smooth muscle cells, podocytes, macrophages, adipocytes, and other inflammatory cells [7], [8], [23], [24], [25]. This widespread distribution enables aldosterone signaling to function as a cross-organ pathobiologic network rather than as an isolated renal sodium-retention mechanism.

In the heart, MR activation promotes myocardial fibrosis, electrical remodeling, and arrhythmogenic substrate formation [23], [26]. In the vasculature, it contributes to endothelial dysfunction, vascular stiffness, and inflammatory activation [12], [25], [27]. In the kidney, MR signaling accelerates podocyte injury and proteinuria progression [8], [28]. In immune and metabolic tissues, aldosterone influences macrophage polarization and adipose inflammation, further amplifying systemic injury pathways [24].

This ubiquity supports a systems-level model in which cumulative aldosterone exposure drives parallel injury across organ systems. Collectively, these observations position MR activation as a central systems-level amplifier of cardiovascular and renal remodeling, reinforcing its candidacy as an upstream therapeutic target.

## 3 Evidence base for MR blockade

### 3.1 Resistant hypertension

PATHWAY-2 established spironolactone as the most effective fourth-line therapy in resistant hypertension [17], [29].

### 3.2 Heart failure (HFrEF)

RALES and EPHESUS demonstrated morbidity and mortality reduction in HFrEF and post-MI LV dysfunction [30], [31], [32].

### 3.3 HFpEF

TOPCAT suggested benefit in selected HFpEF populations [33], [34].

### 3.4 Diabetic CKD

FIDELIO-DKD and FIGARO-DKD demonstrated that finerenone reduced kidney disease progression and cardiovascular events when added to ACE inhibitor/ARB therapy [35], [36], [37], [38], [39]. It should be noted that much of the organ-protective evidence derives from heart failure and diabetic CKD populations, and direct evidence in uncomplicated hypertension with preserved renal function remains limited.

### 3.5 Contemporary context: SGLT2 inhibition

The SGLT2 inhibitor era further reinforces the principle that organ protection may occur via mechanisms partially independent of blood pressure lowering. Large cardiovascular and renal outcome trials have demonstrated reductions in heart failure hospitalization and chronic kidney disease progression with SGLT2 inhibition across diverse populations, including patients with and without diabetes [40], [41]. These benefits appear only modestly correlated with achieved BP reduction, suggesting biologic modulation of cardiorenal injury pathways beyond hemodynamic control.

Emerging analyses suggest complementary and potentially additive effects when SGLT2 inhibitors and MR antagonists are combined, reflecting convergence on overlapping but mechanistically distinct pathways of fibrosis, inflammation, and tubular injury. In this evolving therapeutic landscape, the concept of earlier, risk-stratified MR antagonism aligns with a broader shift in hypertension management—from isolated pressure reduction toward integrated hemodynamic and biologic protection.

### 3.6 Comparative considerations among MRAs

The available MRAs are not interchangeable, and agent selection should be individualized. Spironolactone is a nonselective steroidal MRA and the most extensively validated agent for blood-pressure lowering in resistant hypertension [17], its principal drawbacks being antiandrogenic effects (gynecomastia, menstrual irregularity); it is inexpensive and widely available. Eplerenone is a selective steroidal MRA with fewer sex-hormone-related effects but lower potency and a shorter half-life, with outcome evidence in post-myocardial-infarction left ventricular dysfunction [31]. Finerenone is a selective nonsteroidal MRA with a more balanced cardiac–renal tissue distribution and, in preclinical models, greater anti-inflammatory and anti-fibrotic effect than steroidal agents [39], [42], [43]; it reduces cardiorenal events in diabetic CKD and has demonstrated a manageable hyperkalemia profile under structured monitoring [20], [35], [36], although reliable direct comparisons of hyperkalemia risk with steroidal MRAs remain limited; it is more costly and has not been tested in uncomplicated hypertension with preserved renal function. For the early, prevention-oriented framework proposed here, finerenone is mechanistically attractive, whereas steroidal MRAs remain the established evidence base for resistant hypertension; cost, availability, and the absence of hypertension-specific outcome data are important limitations that reinforce the hypothesis-generating status of this proposal [44].

### 3.7 Positioning within contemporary hypertension guidelines

Current hypertension guidelines emphasize stepwise escalation of therapy based on blood pressure thresholds and overall cardiovascular risk [1], [17]. Mineralocorticoid receptor antagonists are typically reserved for resistant hypertension or for patients with heart failure with reduced ejection fraction. However, these recommendations were largely derived from blood pressure–centric trial designs rather than from studies specifically evaluating early tissue-level biologic protection.

Notably, major hypertension guidelines do not explicitly incorporate biomarkers or mechanistic mediators such as aldosterone–MR activation into first-line risk stratification frameworks. As a result, MR antagonism is often introduced late—after vascular stiffness, renal dysfunction, or myocardial remodeling are already established.

The framework proposed here does not conflict with current guidelines but instead suggests a biologically informed refinement: earlier MR antagonism in carefully selected, high-risk hypertensive phenotypes with preserved renal function and controlled potassium levels.

This represents a potential evolution from purely hemodynamic control toward integrated hemodynamic and biologic modulation.

### 3.8 The therapeutic timing paradox in hypertension: why delayed MRA use increases hyperkalemia fear

**Fig. 3:**
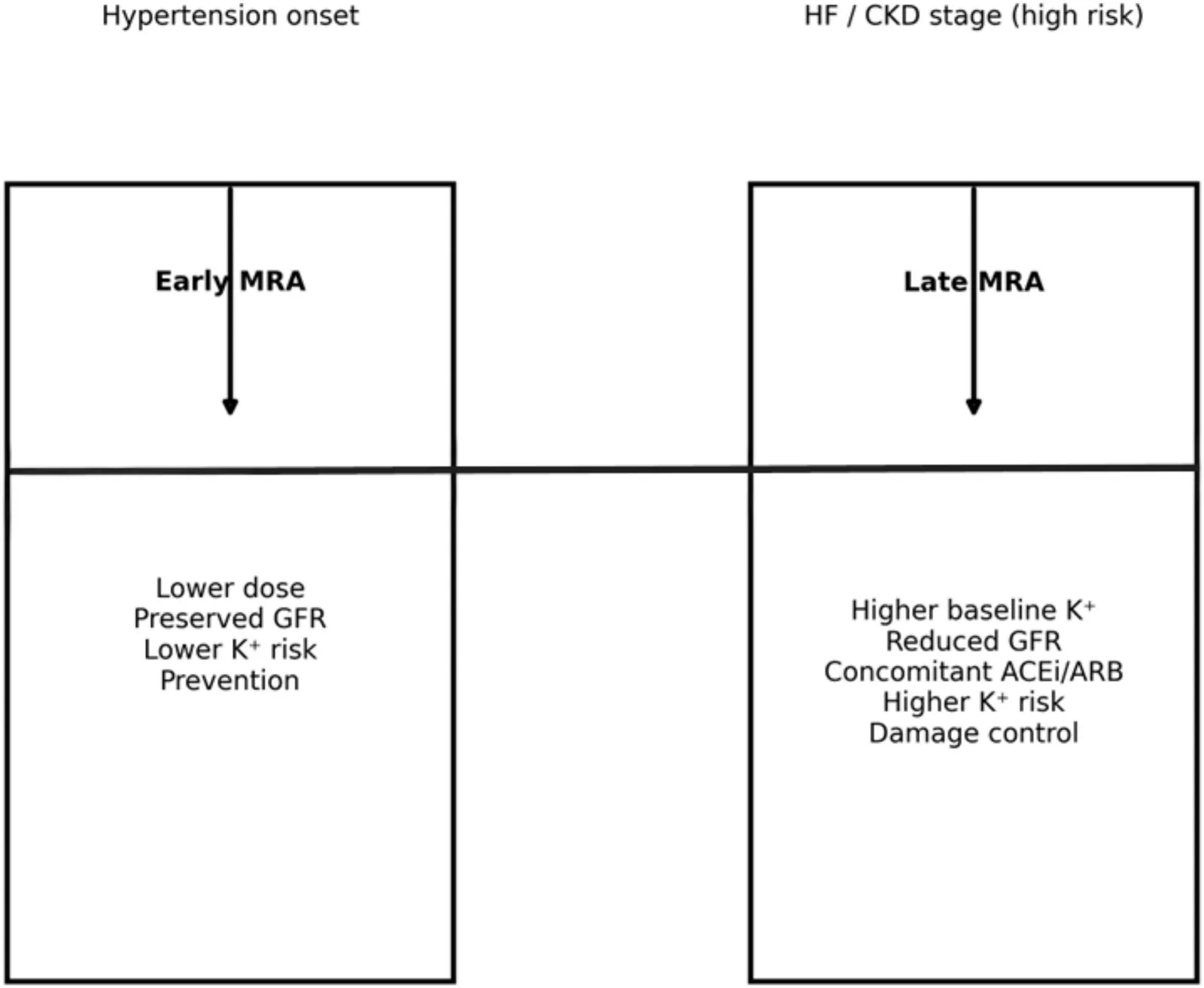
Timing paradox of mineralocorticoid receptor antagonist therapy. Delayed initiation commonly coincides with reduced estimated glomerular filtration rate and concomitant ACE inhibitor or angiotensin receptor blocker therapy, amplifying hyperkalemia concerns and limiting preventive opportunity.

MR antagonists are frequently introduced late in the natural history of hypertensive disease—often after chronic kidney disease progression or heart failure diagnosis—when estimated glomerular filtration rate is reduced and baseline potassium is higher [45], [46], [47]. At this stage, hyperkalemia risk is amplified and clinicians may be reluctant to initiate therapy. This creates a therapeutic timing paradox: MR blockade is commonly introduced when both organ injury is advanced and safety concerns are greatest (Fig. 3).

Across major MRA trials and analyses, participants with better-preserved renal function and lower baseline potassium demonstrated substantially lower hyperkalemia risk [20], [37], [42]. These observations raise the hypothesis that earlier, carefully monitored MR antagonism may represent a lower-risk window for biologic intervention.

### 3.9 Early, risk-stratified MRA integration for end-organ protection

A prevention-oriented perspective considers mineralocorticoid receptor antagonism as a potential adjunctive organ-protective strategy rather than solely as salvage therapy. However, such consideration should remain confined to carefully selected high-risk phenotypes with preserved renal function and controlled potassium levels, and within the boundaries of current guideline-directed therapy. This hypothesis-generating framework does not advocate routine early use, but instead proposes that the timing of MR antagonism may merit prospective evaluation in biologically enriched subgroups. Candidates for earlier consideration include resistant hypertension, albuminuria/early CKD with preserved eGFR, LVH/HFpEF phenotype, and cardiometabolic risk states (diabetes, obesity/OSA) [17], [19], [33], [48], [49]. Guidance recognizes spironolactone for resistant hypertension with renal/potassium caution thresholds [2], [29]. Nonsteroidal MR antagonists (e.g., finerenone) may broaden options in selected populations, though potassium monitoring remains essential [37], [39], [42].

An earlier, risk-stratified integration in selected patients with preserved renal function may:

• Permit lower dosing
• Occur in lower potassium-risk environments
• Extend preventive exposure prior to irreversible remodeling

Importantly, no randomized trial has yet evaluated systematic early MRA initiation in uncomplicated hypertension with preserved renal function. Therefore, this framework is hypothesis-generating and intended to stimulate prospective evaluation rather than alter current guideline indications.

### 3.10 Hyperkalemia: risk stratification and mitigation

Hyperkalemia remains the primary safety concern.

Structured mitigation includes:

• Baseline potassium and eGFR assessment
• Avoidance when eGFR <45 or K > 5.0 mmol/L (context dependent)
• Early recheck at 1–2 weeks
• Repeat at 4–6 weeks
• Periodic 3–6 month monitoring
• Review of potassium-raising medications
• Diuretic optimization

Recent analyses indicate that hyperkalemia risk is strongly stratified by baseline renal function and potassium levels. In preserved renal function populations, event rates are substantially lower under structured monitoring [42], [46], [50]. In large contemporary trials, the incidence of potassium >5.5 mmol/L ranged from approximately 10–18%, while permanent discontinuation due to hyperkalemia was generally below 3%, particularly in patients with preserved renal function [20], [35], [36]. In FIDELIO-DKD, discontinuation due to hyperkalemia occurred in approximately 2% of participants [35]. These data suggest that while biochemical potassium elevations are relatively common, clinically significant events are strongly modulated by baseline renal function and structured monitoring [42], [50].

### 3.11 Practical risk mitigation considerations

In clinical practice, several pragmatic strategies may reduce hyperkalemia risk when MR antagonists are introduced earlier in the disease course. These include low-dose initiation, careful titration, structured potassium monitoring, avoidance of nonsteroidal anti-inflammatory drugs, and dietary counseling regarding potassium intake. Contemporary potassium binders, including patiromer and sodium zirconium cyclosilicate (SZC), may expand the therapeutic window for RAAS- and MR-directed therapies [50]. The two agents are not equivalent: patiromer exchanges potassium primarily for calcium and is sodium-free, whereas SZC preferentially captures potassium in exchange for hydrogen and sodium ions and is associated with a sodium load (approximately 400 mg per 5-g dose). Because this sodium load has been associated with dose-dependent edema and, in observational data, with heart-failure events [51], SZC may be less desirable in the salt-sensitive, volume-expanded phenotype emphasized in this review; patiromer may therefore be the preferred binder when potassium binding is considered in this population [50]. In the DIAMOND trial, patiromer facilitated optimization of RAAS-inhibitor therapy, including MRA use, in patients with HFrEF and current or previous hyperkalemia [52]. Whether either binder would support a preventive early-MRA strategy in hypertension remains unknown.

Importantly, hyperkalemia risk appears strongly modulated by baseline renal function. Patients with better-preserved estimated glomerular filtration rate (eGFR ≥45–60 mL/min/1.73 m^2^) and baseline potassium ≤5.0 mmol/L have demonstrated substantially lower rates of clinically significant hyperkalemia across major MRA trials [30], [31], [35], [36].

These observations provide a biologically plausible rationale that earlier-stage disease may represent a lower-risk window for intervention.

### 3.12 A conceptual research framework (Fig. 4)

**Fig. 4:**
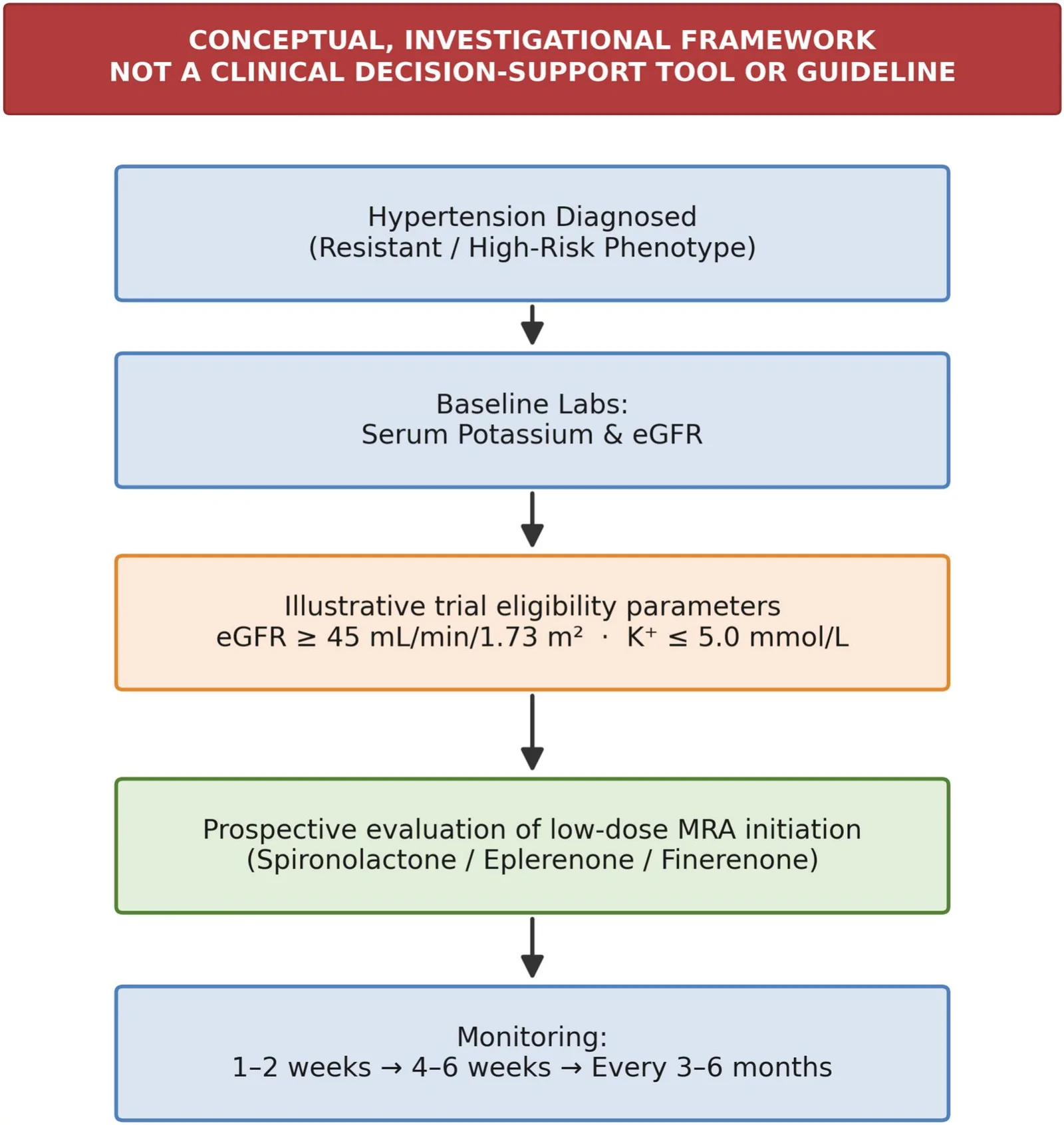
Conceptual research framework for prospectively evaluating early mineralocorticoid receptor antagonist timing and potassium monitoring in hypertension. The phenotypes, eGFR and potassium values, and monitoring intervals shown are illustrative parameters proposed for testing in future trials, not validated eligibility criteria or clinical recommendations. This figure does not constitute clinical guidance and does not replace guideline-directed indications or diagnostic evaluation for primary aldosteronism.

Fig. 4 illustrates a conceptual pathway by which earlier MR blockade could be evaluated in future studies: identification of higher-risk phenotypes (resistant hypertension, albuminuria, LVH/HFpEF, diabetes/OSA/obesity), confirmation of baseline potassium and eGFR, low-dose MRA initiation in eligible participants, and structured monitoring with dose adjustment. It is presented as a hypothesis-generating research framework, not a clinical decision-support tool, and does not advocate deviation from current guideline-directed indications. It is distinct from, and does not replace, established diagnostic pathways for primary aldosteronism.

### 3.13 Adjunctive potassium-mitigation strategies

Thiazide-based therapy and low-dose loop diuretics may theoretically offset potassium elevation in selected patients. While biologically plausible and frequently used in practice, such strategies require prospective validation before incorporation into preventive protocols.

### 3.14 Implications for future clinical evaluation

For clinicians managing hypertension, persistent albuminuria, left ventricular hypertrophy, or cardiometabolic risk may reflect ongoing aldosterone–MR signaling despite numeric BP control [5], [7], [17]. This observation raises a testable hypothesis rather than a treatment recommendation: whether, in carefully selected patients with preserved renal function and normokalemia, earlier low-dose MR antagonism under structured monitoring could reduce tissue-level injury. This question is not yet answered by randomized evidence in uncomplicated hypertension and should be evaluated prospectively within—not ahead of—current guideline-directed frameworks. The framework emphasizes the timing of biologic intervention as a research question, not escalation of therapy in routine practice.

## 4 RAAS modulation: clinical considerations in Black patients

### 4.1 Potentially unopposed aldosterone signaling with commonly used initial antihypertensive therapies

**Fig. 5:**
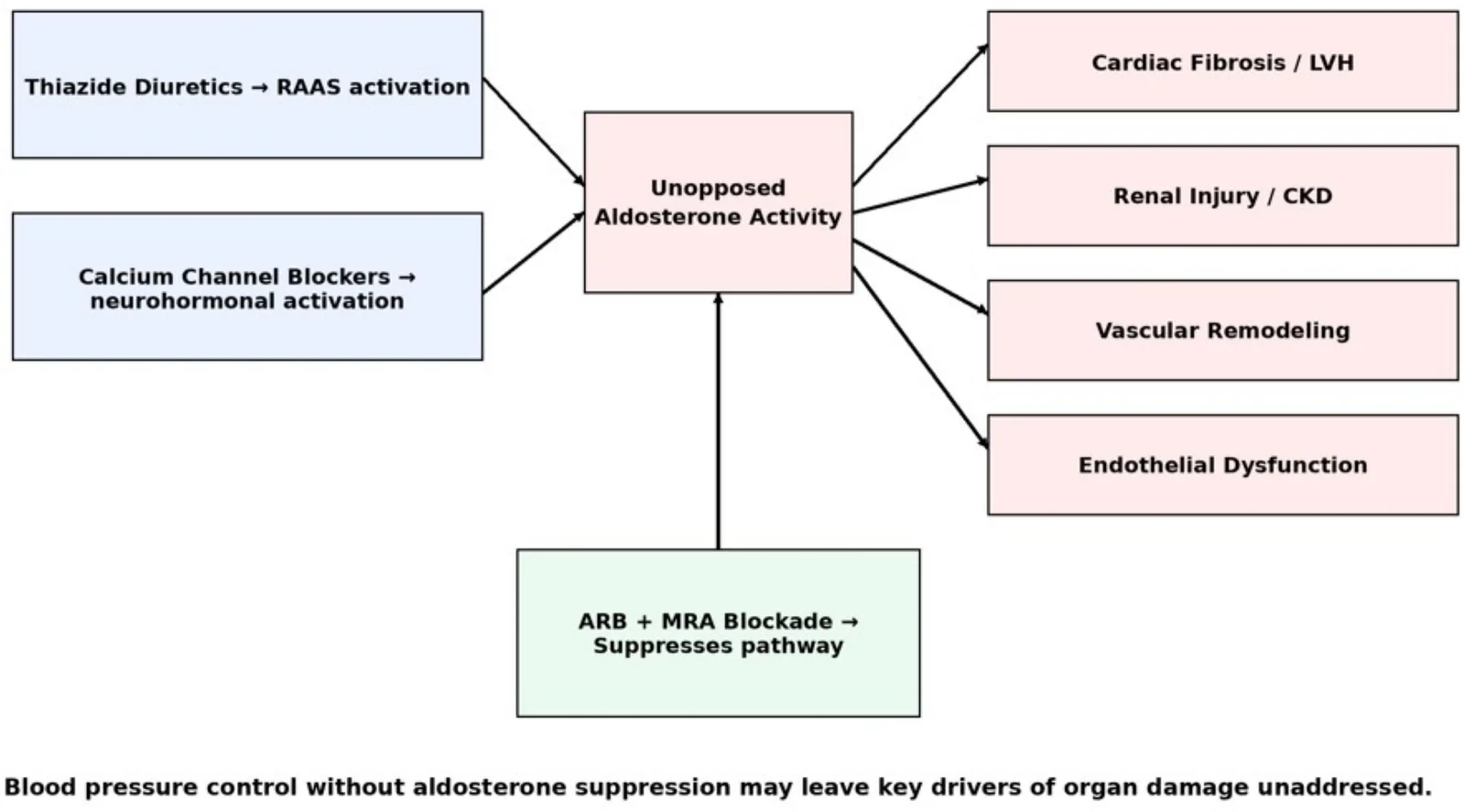
Unopposed aldosterone signaling during thiazide or calcium channel blocker monotherapy without RAAS or MR blockade, and the modifying role of ARBs and MRAs in attenuating these pathways and mitigating downstream tissue effects.

Thiazide diuretics and dihydropyridine calcium channel blockers (CCBs) are effective antihypertensive agents and are frequently used as first-line therapy in African American patients due to favorable blood pressure responses. However, both classes may induce compensatory neurohormonal activation, including stimulation of the renin–angiotensin–aldosterone system (RAAS). In the absence of concurrent RAAS or mineralocorticoid receptor blockade, this compensatory activation may leave aldosterone-mediated pathways of inflammation, fibrosis, and vascular remodeling unopposed (Fig. 5). Persistent mineralocorticoid receptor activation contributes to progressive myocardial, renal, and vascular injury independent of blood pressure control.

Beyond their neurohormonal effects, these agents carry class-specific safety considerations. Thiazide and thiazide-like diuretics may cause hypokalemia, hyponatremia, hyperuricemia and gout, and impaired glucose tolerance, and activate the renin–angiotensin–aldosterone system as a compensatory response [1], [18]. Dihydropyridine calcium-channel blockers commonly cause dose-dependent peripheral edema and reflex neurohormonal activation and do not suppress aldosterone [2]. In each case, effective BP lowering may occur without directly attenuating—and in some settings may be accompanied by compensatory activation of—RAAS/aldosterone signaling, providing a mechanistic rationale for evaluating complementary MR-directed therapy in appropriately selected patients (Fig. 5).

### 4.2 ACE inhibitor versus ARB selection: angioedema risk and implications for future heart-failure therapy

**Fig. 6:**
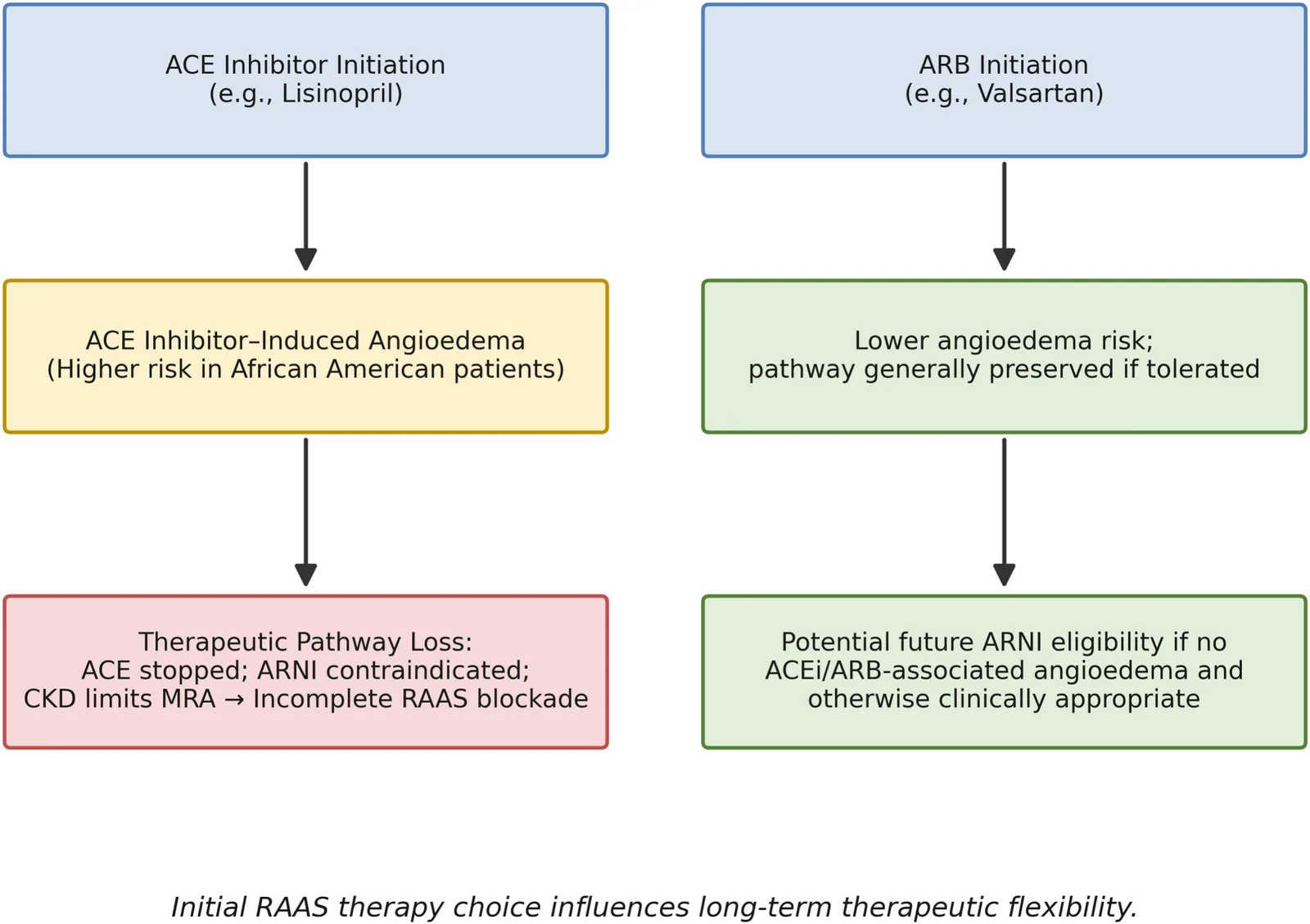
Implications of ACE inhibitor–associated angioedema for subsequent RAAS-directed therapy in African American patients. ACE inhibitor–associated angioedema, which occurs more frequently in African American patients, may limit subsequent therapeutic options, including ARNI use and, in advanced disease, MRA integration. In contrast, an ARB-based strategy may preserve greater therapeutic flexibility when tolerated and when no ARB-associated angioedema occurs.

African American patients have a higher incidence of ACE inhibitor–associated angioedema, a complication that may have long-term therapeutic implications. While angiotensin receptor blockers (ARBs) may often be used cautiously in such patients, the occurrence of ACE inhibitor–associated angioedema generally precludes subsequent use of sacubitril/valsartan, because a history of angioedema related to prior ACE inhibitor or ARB therapy is a contraindication, thereby potentially limiting access to a key component of guideline-directed heart failure therapy [53]. As renal dysfunction progresses, the use of mineralocorticoid receptor antagonists (MRAs) may also be constrained by hyperkalemia risk. This sequence of events may result in incomplete neurohormonal blockade at advanced stages of disease, when comprehensive pathway inhibition is most critical (Fig. 6).

Together, these clinical observations suggest that both unopposed aldosterone signaling (Fig. 5) and early therapy-related pathway restrictions (Fig. 6) may contribute to preventable progression of end-organ damage in high-risk, medically underserved populations.

### 4.3 Knowledge gaps and future directions

Despite mechanistic and clinical convergence, key questions remain:

• Does early low-dose MRA reduce incident HFpEF or atrial fibrillation?
• Can structured potassium mitigation safely expand eligibility?
• Which phenotypic subgroups derive maximal preventive benefit?
• What biomarkers best reflect early aldosterone-driven tissue injury?

Therefore, prospective randomized trials evaluating early MRA integration in hypertensive populations with preserved renal function are needed to define risk–benefit balance and inform future guideline evolution.

### 4.4 Conceptual trial design considerations

The future randomized trial testing early MR antagonism in hypertension might enroll patients with newly diagnosed or high-risk stage 1–2 hypertension, preserved renal function, and normokalemia. Mechanistically aligned endpoints—such as progression of albuminuria, left ventricular mass index, or incident atrial fibrillation—may more accurately reflect tissue-level protection than blood pressure reduction alone.

Finerenone trials in diabetic CKD enrolled carefully monitored participants with eGFR values as low as 25 mL/min/1.73 m^2^ and generally required serum potassium no higher than 4.8 mmol/L at screening [35], [36], [37]. The higher potassium thresholds used during treatment represented dose-interruption criteria rather than entry criteria. For the preventive framework proposed here, an illustrative eGFR threshold of ≥45 mL/min/1.73 m^2^ is intentionally conservative and is intended to define a lower-risk research population. Future trials with intensive monitoring could separately evaluate an eGFR boundary of ≥30 mL/min/1.73 m^2^.

### 4.5 Limitations of the proposed framework

The conceptual model proposed herein warrants several important limitations. First, although robust outcome data support mineralocorticoid receptor antagonists in resistant hypertension, heart failure, and diabetic chronic kidney disease, no randomized trial has specifically evaluated systematic early initiation of MR antagonism in uncomplicated stage 1–2 hypertension with preserved renal function. Therefore, the preventive timing hypothesis remains inferential and extrapolated from adjacent high-risk populations.

Second, while hyperkalemia risk appears strongly stratified by baseline renal function and potassium levels, structured mitigation strategies—including diuretic co-therapy or potassium binders—require prospective validation before incorporation into preventive protocols. Third, biomarkers that best identify early aldosterone-driven tissue injury have not yet been standardized for clinical decision-making. Finally, broader application of MR antagonists in lower-risk hypertensive populations could introduce potential overtreatment or cost-effectiveness considerations that must be carefully studied.

Accordingly, this framework is intended to stimulate mechanistically aligned clinical investigation rather than to alter current guideline-directed therapy.

These considerations may be particularly relevant in populations disproportionately affected by hypertension-related morbidity. Earlier identification of aldosterone-mediated injury and risk-stratified MR antagonism may represent an opportunity to mitigate long-standing disparities in cardiovascular and renal outcomes.

## 5 Conclusion

Hypertension management requires both effective BP control and attention to biologic pathways that drive tissue injury. Aldosterone–MR signaling appears to function as a central pathophysiologic effector of fibrosis, inflammation, and adverse remodeling across vascularized organs.

While current evidence robustly supports MRAs in resistant hypertension, heart failure, and diabetic CKD, emerging biologic insights justify prospective evaluation of earlier, risk-stratified integration in selected populations under structured safety monitoring.

Pairing hemodynamic pressure control with earlier biologic modulation of mineralocorticoid receptor signaling may represent a rational next step in the evolution of hypertension management. This approach may be particularly relevant for reducing disproportionate cardiovascular and renal outcomes in populations historically burdened by hypertension-related disease.

## Declaration of generative AI and AI-assisted technologies in the manuscript preparation process

During the preparation of this work the author used ChatGPT to edit, refine, and structure the manuscript. After using this tool, the author reviewed and edited all content, independently verified every reference against primary sources and removed any citation that could not be confirmed, and takes full responsibility for the scientific interpretation, conclusions, and content of the published article, which reflect the author's own original analysis.

## CRediT authorship contribution statement

**Camellus O. Ezeugwu:** Conceptualization, Methodology, Investigation, Writing – original draft, Writing – review & editing, Visualization.

## Funding

This research did not receive any specific grant from funding agencies in the public, commercial, or not-for-profit sectors.

## **Declaration of competing interest**

The author reports no relevant financial disclosures or conflicts of interest.

## Data Availability

This is a review article. No new data were created or analyzed. All data discussed are available within the cited published literature.
